# Role of Macrophages in Cytotoxicity, Reactive Oxygen Species Production and DNA Damage in 1,2-Dichloropropane-Exposed Human Cholangiocytes In Vitro

**DOI:** 10.3390/toxics9060128

**Published:** 2021-06-01

**Authors:** Abigail Ekuban, Cai Zong, Frederick Adams Ekuban, Yusuke Kimura, Ryoya Takizawa, Kota Morikawa, Kazuo Kinoshita, Sahoko Ichihara, Seiichiroh Ohsako, Gaku Ichihara

**Affiliations:** 1 Department of Occupational and Environmental Health, Tokyo University of Science, Noda 278-8510, Japan; 3B18703@alumni.tus.ac.jp (A.E.); zongcai@rs.tus.ac.jp (C.Z.); 3B18701@alumni.tus.ac.jp (F.A.E.); 3a12042@alumni.tus.ac.jp (Y.K.); ryoya.2008.0709@docomo.ne.jp (R.T.); mkouta9331@gmail.com (K.M.); 2 Department of Environmental and Preventive Medicine, Jichi Medical University School of Medicine, Shimotsuke 329-0498, Japan; saho@jichi.ac.jp; 3 Evolutionary Medicine, Shizuoka Graduate University of Public Health, Shizuoka 420-0881, Japan; kkinoshi@mac.com; 4 Laboratory of Environmental Health Sciences, Faculty of Medicine, University of Tokyo, Tokyo 113-8655, Japan; ohsako@m.u-tokyo.ac.jp

**Keywords:** 1,2-Dichloropropane, cytotoxicity, cholangiocarcinoma, chemical carcinogenesis, work safety, carcinogenic compounds, inflammation-related carcinogenesis, macrophages

## Abstract

1,2-Dichloropropane (1,2-DCP), a synthetic chlorinated organic compound, was extensively used in the past in offset color proof-printing. In 2014, the International Agency for Research on Cancer (IARC) reclassified 1,2-DCP from its initial Group 3 to Group 1. Prior to the reclassification, cholangiocarcinoma was diagnosed in a group of workers exposed to 1,2 -DCP in an offset color proof-printing company in Japan. In comparison with other forms of cholangiocarcinoma, 1,2-DCP-induced cholangiocarcinoma was of early onset and accompanied by extensive pre-cancerous lesions in large bile ducts. However, the mechanism of 1,2-DCP-induced cholangiocarcinoma is poorly understood. Inflammatory cell proliferation was observed in various sites of the bile duct in the noncancerous hepatic tissues of the 1,2-DCP-induced cholangiocarcinoma. The aim of this study was to enhance our understanding of the mechanism of 1,2-DCP-related cholangiocarcinogenesis. We applied an in vitro system to investigate the effects of 1,2-DCP, using MMNK-1 cholangiocytes cultured alone or with THP-1 macrophages. The cultured cells were exposed to 1,2-DCP at 0, 0.1, 0.2, 0.4, and 0.8 mM for 24 h, and then assessed for cell proliferation, cell cytotoxicity, DNA damage, and ROS production. Exposure to 1,2-DCP increased proliferation of MMNK-1 cholangiocytes cultured alone, but not those cultured with macrophages. 1,2-DCP also increased LDH cytotoxicity, DNA damage, and ROS production in MMNK-1 cholangiocytes co-cultured with macrophages but not those cultured alone. 1,2-DCP increased TNFα and IL-1β protein expression in macrophages. The results highlight the role of macrophages in enhancing the effects of 1,2-DCP on cytotoxicity, ROS production, and DNA damage in cholangiocytes.

## 1. Introduction

1,2-Dichloropropane (1,2-DCP) is a synthetic chlorinated volatile organic compound mostly used as a chemical intermediate in the production of other organic solvents, such as propylene, carbon tetrachloride, and tetrachloroethylene. It is used in solvent-based degreasers, cleaning products, coating products, adhesives, and sealants [1,2]. Moreover, 1,2-DCP has been used as an ink-removing agent in the printing industry, and human exposure has been shown to occur mainly in occupational settings [2]. 1,2-DCP was reclassified in 2014 by the International Agency for Research on Cancer (IARC) from Group 3 (not classifiable as to its carcinogenicity to humans) to Group 1 (carcinogenic to humans) [2]. Before the above revision of 1,2-DCP classification, several cases of cholangiocarcinomas were reported among workers of an offset color proof-printing section of a printing company in Japan. Most of those affected were exposed to 1,2-DCP when they removed ink from the transcription rubber roller [3]. The initial cluster of cholangiocarcinoma cases was first found in a printing company in Osaka, where 17 workers between the ages 25 and 45 years were diagnosed [4,5]. Further investigations identified more cases of cholangiocarcinomas associated with chemical exposure in other printing companies [6,7]. The Ministry of Health, Labor, and Welfare of Japan labelled these cholangiocarcinomas as an occupational disease [8]. Cholangiocarcinoma in these workers occurred at a younger age compared to overall cholangiocarcinoma registered regionally [4]. Histopathological examination of sections from the surgically-excised tumors obtained from workers exposed to 1,2-DCP showed precancerous or early cancerous lesions, such as biliary intraepithelial neoplasia (BilIN) and intraductal papillary neoplasm of the bile duct (IPNB), together with sclerosis of the bile duct, inflammatory cell infiltration, biliary epithelial injuries/focal bile duct loss, and biliary epithelial hyperplasia at various areas of the bile ducts in the noncancerous hepatic tissues [4]. Immunohistochemical analysis showed over-expression of γ-H2AX, a marker of DNA double strand break, in the foci of BilIN, IPNB, invasive carcinoma, and non-neoplastic biliary epithelial cells, compared to specimens from control cholangiocarcinomas [9]. Whole-exome analysis of cholangiocarcinoma obtained from workers exposed to 1,2-DCP showed a 30-fold higher rate of somatic mutations compared with common cholangiocarcinoma and unique trinucleotide mutational change [10].

Nonetheless, the mechanisms of 1,2-DCP-induced cholangiocarcinogenesis, remains poorly understood. Contrary to the aforementioned cases in humans, the evidence of cholangiocarcinoma caused by 1,2-DCP has not been experimentally established [11]. Available data on animal studies show that 1,2-DCP was not carcinogenic to the bile duct in mice or rats. In an oral gavage study in mice and rats, exposure to 1,2-DCP induced hepatocellular carcinoma and no exposure-related effects, respectively [12]. Moreover, inhalation exposure to 1,2-DCP was shown to induce nasal tumors in a rat carcinogenicity study [13]. Furthermore, oral exposure to 1,2-DCP by gavage was not found be cholangiocarcinogenic in hamsters [11].

It is well known that dihalogenated hydrocarbons, such as ethylene dibromide and ethylene dichloride, produce episulfonium ion, which forms DNA adduct through glutathione conjugation [14]. However, one in vivo study could not confirm the formation of episulfonium ion from 1,2-DCP [15]. Previous studies on the genotoxicity of 1,2-DCP yielded mixed results of either positive or negative for both in vitro and in vivo assessment [16,17,18,19,20]. Although 1,2-DCP was found not to be mutagenic in the Ames assay test using *Salmonella typhimurium* strains TA1537, TA1538, TA98, and TA1978 [16,19,21], a recent study reported a dose-dependent mutagenic activity in the TA100 strain [10]. 1,2-DCP was not positive in the dominant-lethal assay conducted by the United States Environmental Protection Agency (USEPA), in which Sprague-Dawley rats were exposed to 1,2-DCP through drinking water [22]. However, in an in vitro study, exposure to 1,2-DCP induced sister chromatid exchange and chromosomal aberration in Chinese hamster ovary cells, both with and without exogenous metabolic activation [17]. Thus, there is no direct evidence for the formation of DNA adducts by 1,2-DCP or its metabolites, and it is still unclear how 1,2-DCP induces the DNA damage observed in specimens from the workers exposed to 1,2-DCP.

Certain chemicals exhibit enhanced toxicity in the presence of inflammatory cells [23]. Various studies have demonstrated activation of infiltrating macrophages following exposure of laboratory animals to toxicants, and also that these cells contribute to liver injury [24,25]. Furthermore, oral treatment of experimental animals with 1,2-DCP is followed by inflammatory cell infiltration in the liver parenchyma [26]. Moreover, inflammatory cell proliferation was observed in the bile duct in the noncancerous hepatic tissues of the aforementioned occupational cholangiocarcinoma cases [4]. We have also demonstrated recently that exposure of human cholangiocytes co-cultured with macrophages to 1,2-DCP induced the expression of activation-induced cytidine deaminase (AID), which is known to be upregulated by proinflammatory cytokines, such as IL-1β or TNFα, in a variety of tissues [27].

Reactive oxygen species (ROS) are widely implicated in various pathogenic processes, including chemical carcinogenesis [28,29]. More specifically, ROS are considered to play a part in the enhanced toxicity of various chemicals observed in the presence of inflammatory cells [30,31,32]. In addition, the metabolism of xenobiotics is known to generate ROS [33,34,35].

The aim of the present study was to better understand the molecular mechanisms of 1,2-DCP-induced cholangiocarcinogenesis. Towards this goal, we determined the effects of exposure to 1,2-DCP on cell survival, DNA damage, and ROS production in cholangiocytes cultured alone or in the presence of macrophages.

## 2. Materials and Methods

### 2.1. Cell Lines and Cell Cultures

Human immortalized cholangiocytes (MMNK-1 cells) were obtained from Japan Collection of Research Bioresources Cell Bank (JCRB, Osaka, Japan). The cell line expresses cholangiocyte markers such as cytokeratin (CK)-7 and CK-19, and also exhibits cholangiogenic tubule formation in a Matrigel assay [36]. MMNK-1 cells were maintained in Dulbecco’s modified eagle medium, low glucose (DMEM, Wako Pure Chemical Industries, Osaka, Japan) and supplemented with 5% heat-inactivated fetal bovine serum (FBS, Biowest, lot. no. S17692S1820, Riverside, MO, USA) at 37 °C under an atmosphere of 5% CO_2_. MMNK-1 cells were detached by Accutase (Innovative Cell Technologies, San Diego, CA, USA) and sub-cultured every 2–3 days.

Human monocytic cells (THP-1 cells) obtained from the American Type Culture Collection (ATCC, Rockville, MD, USA). THP-1 cells express Fc and C3b receptors, and regarding human lymphocyte antigen (HLA) typing, the cells possess HLA-A2, -A9, -B5, -DRW1, and -DRW2 histocompatibility antigens [37].They were maintained in Roswell Park Memorial Institute medium 1640 (RPMI1640, Wako, Japan) supplemented with 10% heat-inactivated FBS, penicillin, streptomycin, L-glutamine (Gibco, Thermo Fisher, Waltham, MA, USA), and 2-mercapethanol (0.05 mM, Sigma Aldrich, St. Louis, MO, USA), at 37 °C in an atmosphere of 5% CO_2_. THP-1 cells were passaged every 3 days, and passage numbers between 12 and 18 were used for the experiments in the present study. THP-1 cells were differentiated into macrophages by treatment with 162 nM phorbol 12-myristate 13-acetate (PMA, Sigma-Aldrich) dissolved in the complete medium for THP-1 cells, over a period of 48 h, at 37 °C in an atmosphere of 5% CO_2_ [38].

All seeding densities for the various research methods used are stated in Table 1.

### 2.2. Co-Culture Method

Firstly, THP-1 cells treated with 162 nM PMA were seeded into cell culture inserts with membrane pore size 0.4 µm (Corning, Kennebunk, ME, USA) and incubated at 37 °C in an atmosphere of 5% CO_2_ for 48 h (Supplementary Figure S1). The inserts were then washed three times with phosphate-buffered saline (PBS) and incubated at 37 °C in an atmosphere of 5% CO_2_ in fresh complete medium for THP-1 cells, for 4–5 h. Secondly, MMNK-1 cells were seeded in well plates and cultured for 12 h, then co-cultured with the 48-h-differentiated THP-1 macrophages, for an additional period of 12 h in a mixture of DMEM and RPMI 1640 of 1:1 ratio, supplemented with 5% FBS. This was followed by 1,2-DCP exposure at different concentrations for 24 h as described previously [27].

### 2.3. Determination of 1,2-DCP Concentrations

The estimated exposure concentrations of 1,2-DCP in workers who developed cholangiocarcinoma ranged from 100 to 670 ppm [3]. The following assumptions were taken into consideration to determine the required 1,2-DCP exposure level in ppm (*v*/*v*) that matches the concentration of 1,2-DCP in human blood. Based on an air partition coefficient of 10.7 [2,39], 1000 ppm (0.22 ppm = 1 mg/m^3^) of inhaled 1,2-DCP is in equilibrium with approximately 0.4 mM of 1,2-DCP in blood. Hence, we used 1,2-DCP at 0.1 to 0.8 mM concentrations in the present study.

### 2.4. Preparation of 1,2-DCP Exposure Solution

1,2-DCP (98% purity) was purchased from Tokyo Chemical Industry (TCI, Tokyo, Japan) and dissolved in dimethyl sulfoxide (DMSO, Wako, Japan). It was subsequently diluted in complete medium for MMNK-1 cells, differentiated THP-1 cells, or co-cultured MMNK-1 with differentiated THP-1 cells. The DMSO concentration was adjusted to 0.1%.

### 2.5. Cell Exposure to 1,2-DCP

The seeded cells were exposed to different concentrations of 1,2-DCP (0, 0.1, 0.2, 0.4, and 0.8 mM) for 24 h at 37 °C, sealed in Tedlar polyvinyl fluoride (PVF) gas sampling bags, as described in detail previously with minor modification [27].

### 2.6. Assessment of Cell Proliferation

Following exposure to 1,2-DCP, cell proliferation was assessed by MTS Assay (CellTiter 96^®^ AQueous One Solution Cell Proliferation Assay, Promega, Madison, WI, USA), cell count (Neubauer Improved Hemocytometer, Erma, Tokyo), and BrdU assay (5-bromo-2′-deoxy-uridine labelling and detection kit II, Roche Diagnostics Co., Indianapolis, IN, USA).

### 2.7. MTS Assay

MTS cell viability assay was conducted for both monocultured MMNK-1 cells and MMNK-1 cells co-cultured with THP-1-derived macrophages. After seeding, the cells were incubated at 37 °C under 5% CO2 overnight (monocultured MMNK-1 cells) or 24 h (co-cultured cells), and then exposed to different concentrations of 1,2-DCP for 24 h. Following 24 h of 1,2-DCP exposure, the MTS assay was performed using CellTiter 96^®^ AQueous One Solution Cell Proliferation Assay (Promega) according to the instructions provided by the manufacturer and as described previously [40]. Absorbance was measured at 490 nm using a microplate reader (PowerWave XS2, BioTek, Winooski, VT, USA). Cell viability at each concentration of 1,2-DCP was expressed as percentage of the absorbance in relation to the absorbance of the corresponding 0 mM 1,2-DCP group either in monoculture or co-culture (cell viability (% of control) = (absorbance of 1,2-DCP—absorbance of blank)/(absorbance of control—absorbance of blank) * 100%).

### 2.8. BrdU Assay

BrdU immunocytochemistry was performed using a 5-bromo-2′-deoxyuridine labelling and detection kit II (Roche) and a mouse-and-rabbit-specific HRP/DAB (ABC) Detection IHC kit (ab64264 -Abcam) following the manufacturer’s protocol with minor modifications as previously described [41]. The seeded MMNK-1 cells were exposed to 1,2-DCP at 0, 0.1, 0.2, 0.4, and 0.8 mM for 24 h. Following exposure, the medium was aspirated and replaced with BrdU labelling medium, and then incubated for 45 min at 37 °C under 5% CO_2_. The cells were fixed with ethanol-based fixative (70% absolute ethanol, 50 mM glycine) at −20 °C for 30 min and blocked with 30% hydrogen peroxide (H_2_O_2_) for 10 min. Unspecific binding proteins were also blocked with 1% bovine serum albumin (BSA) dissolved in PBS for 10 min and incubated for 30 min at 37 °C with anti-BrdU monoclonal antibody. Subsequently, the cells were incubated with biotinylated goat anti-polyvalent antibody for 10 min and further incubated with streptavidin peroxidase for 10 min at room temperature. The cells were then stained with the chromogen and diaminobenzidine (DAB) peroxidase substrate, and counterstained with hematoxylin. The stained cells were mounted with an aqueous mounting medium (Vecta Mount Mounting Medium, H-5501, Vector Laboratories). Images were visualized with an Olympus BX50 microscope (Olympus, Tokyo, Japan) equipped with a Leica color digital camera (Leica DFC 290 HD). Photomicrographs of 10 fields per well were taken and quantified as relative positive stain by counting the number of positively stained nuclei to the total number of nuclei. The total number of nuclei counted per well ranged from 2000 to 3000. BrdU labelling index was calculated by the following equation: BrdU labelling index = relative positive stain of 1,2-DCP/relative positive stain of control * 100.

### 2.9. Cell Count

To assess cell proliferation by cell count, the seeded MMNK-1 cells were exposed to 1,2-DCP at 0, 0.1, 0.2, 0.4, and 0.8 mM concentrations for 24 h. Following the exposure, the cells were detached using Accutase (Innovative Cell Technologies, San Diego, CA, USA). Briefly, aliquot of the cell suspension was diluted in a ratio of 1:1 (*v*/*v*) with 0.5% trypan blue dye, and viable cells were counted using the Neubauer Improved Hemocytometer (Erma) as previously described [42]. The cell count (% of control) at each concentration of 1,2-DCP was expressed as percentage in relation to the cell count at the corresponding 0 mM DCP group, as calculated by the following equation: cell count (% of control) = cell count of 1,2-DCP treated cells/cell count of 0 mM 1,2 DCP treated cells * 100.

### 2.10. LDH Cytotoxicity Assay

Cytotoxicity was assessed by Pierce LDH Cytotoxicity Assay Kit (Pierce, Rockford, IL, USA), which measures LDH activity in the culture media. This was done using the manufacturer’s protocol and as previously described with minor modifications [43]. Seeded monocultured MMNK-1 cells and co-cultures of THP-1 and MMNK-1 cells were exposed to 1,2-DCP at 0, 0.1, 0.2, 0.4, and 0.8 mM for 24 h. LDH activity in the MMNK-1 cells treated with lysis buffer was used as the positive control. Following exposure of the cells, the LDH assay was performed according to the instructions provided by the manufacturer. Absorbance was measured at 490 nm and 680 nm using a microplate reader (PowerWave XS2, BioTek). LDH cytotoxicity at each concentration of 1,2-DCP was expressed as percentage of the absorbance in relation to the absorbance of the corresponding positive control either in monoculture or co-culture by the following equation: cell cytotoxicity (% of positive control) = (absorbance of 1,2-DCP-exposed cells—absorbance of blank)/(absorbance of positive control—absorbance of blank) * 100.

### 2.11. γH2AX Immunofluorescence

Following their exposure to 1,2-DCP, monocultured MMNK-1 cells and MMNK-1 cells co-cultured with THP-1 macrophages were washed in PBS and then fixed in 4% paraformaldehyde for 10 min at room temperature. The cells were then washed thrice in PBST, permeabilized with 0.2% Triton™ X-100 (Sigma Aldrich, St. Louis, MO, USA) in PBS, then blocked with 1% BSA and 22.52 mg/mL glycine in PBST for 30 min, followed by an overnight incubation at 4 °C with the primary antibody, mouse anti-γ-H2AX (dilution, 1:150; sc-517348, Cell Biolabs, San Diego, CA, USA). The cells were then washed thrice and incubated with donkey anti-mouse IgG H&L—Alexa Fluor 647 secondary antibody (dilution, 1:400; ab150111, Abcam, Cambridge, MA, USA)—for one hour at room temperature in the dark. After this, the cells were covered with a fluoroshield mounting medium containing DAPI (ab104139, Abcam, Cambridge, MA, USA). γH2AX staining was visualized using a fluorescent microscope (Leica DMI6000B-AFC, Wetzlar, Germany). The procedure was carried out as described previously [44] with minor modifications. Briefly, photomicrographs of 12 fields per well were taken, and a total of 500 to 2000 cells per well were quantified. γH2AX foci count was determined by calculating the average of γH2AX foci per nucleus with the total number of nuclei. Image J software was used in counting the number of foci per nucleus [45]. Cells exposed to 0.2 mM H_2_O_2_ for 2 h were used as positive control.

### 2.12. Alkaline Comet Assay

Comet slides were pre-coated with 1% normal-melting-point agarose and dried at 60 °C. After their exposure to 1,2-DCP, the cells were washed in cold PBS, centrifuged, and then suspended in 0.75% low-melting-point agarose at a ratio of 1:10 (*v*/*v*) in a single cell suspension. Next, 100 µL of the single cell suspension was pipetted onto the comet slides and kept at 4 °C in the dark for 20 min. The cells were then treated with pre-chilled lysis buffer (2.5 M NaCl, 100 mM EDTA, 10 mM Tris-HCl, pH > 10, 1% *N*-lauroylsarcosine, 1% Triton X-100, 10% DMSO) at 4 °C for 1 h and then in pre-chilled alkaline solution (300 mM NaOH, pH > 13, 1 mM EDTA) for 30 min at 4 °C in the dark. Alkaline electrophoresis was performed at 17V with current set at 200 mA for 15 min. The slides were then washed thrice in pre-chilled distilled water, fixed in 70% ethanol, dried at 37 °C in the dark, and then stained with vista green DNA dye (Cell Biolabs, Inc., San Diego, CA, USA) for 15 min at room temperature. The slides were rinsed briefly in distilled water and dried at 37 °C for an hour as described previously [46]. They were examined under a fluorescent microscope (Leica DMI6000B-AFC, Wetzlar) and images were taken. At least 100 cells per slide were analyzed using Comet Score V2.0 (TriTek Corp., Sumerduck, VA, USA). The positive control cells were exposed to 0.2 mM of H_2_O_2_ for 2 h.

### 2.13. ROS Detection

ROS were detected using the DCFDA Cellular ROS Detection Assay Kit (ab113851, Abcam, Cambridge, MA, USA) and the protocol supplied by the manufacturer, with minor modifications. 1,2-DCP-exposed monocultured MMNK-1 cells, monocultured THP-1 macrophages, and co-cultured MMNK-1 cells with THP-1 macrophages were washed with pre-warmed 1x buffer and stained with 25 µM of DCFDA solution for 45 min in the dark at 37 °C. The DCFDA solution was then removed, and pre-warmed 1x buffer was added. Fluorescence was measured at an excitation wavelength of 485 nm and emission wavelength of 535 nm on a fluorescent plate reader (Wallac 1420 ARVO MX Multilabel Counter, PerkinElmer, Waltham, MA), as carried out previously [47]. ROS production (% of control) was expressed as percentage of the fluorescence in relation to the fluorescence at the corresponding 0 mM 1,2-DCP group either in monoculture or co-culture by the following equation: ROS (% of control) = (fluorescence of 1,2-DCP-exposed cells—fluorescence of blank)/(fluorescence of 0 mM 1,2-DCP-exposed cells—fluorescence of blank) * 100.

### 2.14. ELISA for Proinflammatory Cytokines

TNF-α and IL-1β protein concentrations were quantified using Levis^®^ Human TNF-α ELISA Kit (FUJIFILM Wakoshibayagi Co., Osaka, Japan) and IL-1 beta Human ELISA Kit (Invitrogen, ThermoFisher Scientific, Vienna, Austria), respectively. Differentiated THP-1 cells were exposed to 1,2-DCP at 0, 0.2, 0.4, and 0.8 mM for 24 h. Following the exposure, the cell culture media were collected and centrifuged at 1500 rpm for 10 min at 4 °C, and the supernatants were used for the ELISA test according to the protocol supplied by the manufacturer. Absorbance was measured at 450 nm and 620 nm using a microplate reader (PowerWave XS2, BioTek) as described previously [48]. The protein concentrations were calculated using the absorbance based on the regression equation with either TNF-α or IL-1β standard provided in the respective kit, and expressed as picograms per milliliter (pg/mL). The respective protein concentration values for TNF-α or IL-1β are shown in Supplementary Table S1.

### 2.15. Statistical Analysis

Data were expressed as mean ± standard deviation (SD). One-way analysis of variance (ANOVA) or two-way ANOVA followed by Dunnett’s multiple comparison test (2-tailed) was used for statistical comparisons. Simple regression analysis was used as a trend test with 1,2-DCP exposure level in the presence or absence of macrophages. Multiple regression analysis with full model was conducted to test the interaction of 1,2-DCP exposure level and macrophage factor. When the interaction was not significant, multiple regression analysis in a model without interaction was conducted to test the effect of 1,2-DCP exposure level and that of macrophages. A *p* value less than 0.05 was considered to denote the presence of statistical significance. GraphPad Prism software (version 8.4.2 for Windows, GraphPad Software, San Diego, CA, USA) was used for all analyses, except for the regression analysis, which was performed using JMP software version 15 (SAS Institute, Cary, NC, USA).

## 3. Results

### 3.1. 1,2-DCP Increases Cell Proliferation of Monocultured Cells but Not Co-Cultured Cells

**Figure 1:**
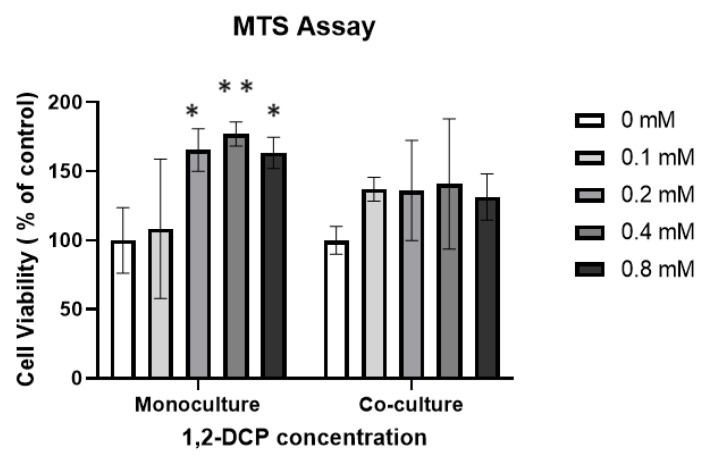
Results of the MTS assay on the effects of 1,2-DCP on cell viability of monocultured MMNK-1 cholangiocytes and MMNK-1 cholangiocytes co-cultured with THP-1 macrophages. Monocultured MMNK-1 cells or co-cultures of MMNK-1 cells/THP-1 macrophages were exposed to 1,2-DCP at 0, 0.1, 0.2, 0.4, and 0.8 mM for 24 h. Percentages are in relation to 0 mM 1,2-DCP group. Data are mean ± SD, *n* = 3. * *p* < 0.05, ** *p* < 0.01 compared with the control group (0 mM), by two-way ANOVA followed by Dunnett’s multiple comparison test.

**Figure 2:**
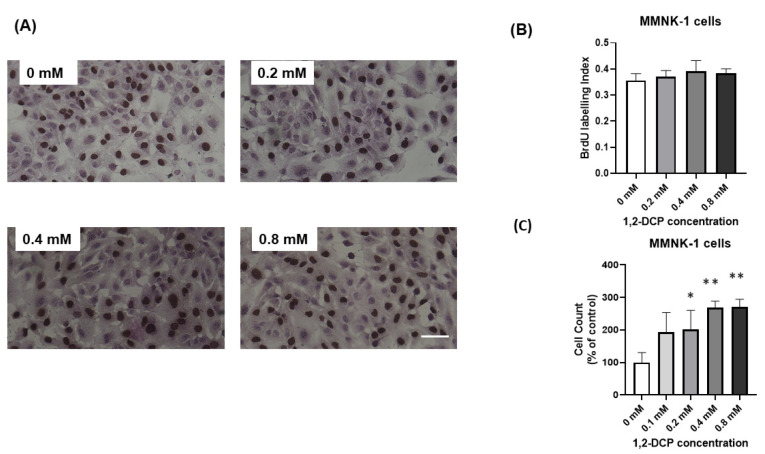
Effects of 1,2-DCP on cell proliferation of monocultured MMNK-1 cholangiocytes. Monocultured MMNK-1 cells were exposed to 1,2-DCP at 0, 0.1, 0.2, 0.4, and 0.8 mM for 24 h. (**A**) Photomicrographs of BrdU immunocytochemistry. MMNK-1 cells were incubated with BrdU labelling medium for 45 min. The cells were washed, fixed with ethanol, blocked with hydrogen peroxide and 1% BSA in PBS, and incubated with anti-BrdU antibody, then with the secondary antibody. The brown color was developed using DAB and nuclei were counterstained with hematoxylin. Cells were observed under a light microscope. Scale bar = 100 µm. (**B**) BrdU labelling index (ratio of the number of BrdU-positive nuclei to the total number of nuclei). (**C**) Cell count (Percentages are in relation to 0 mM 1,2-DCP group). Data are mean ± SD, *n* = 3. * *p* < 0.05, ** *p* < 0.01 compared with the control group (0 mM), by one-way ANOVA followed by Dunnett’s multiple comparison test.

Monocultured MMNK-1 cells and MMNK-1 cells co-cultured with differentiated THP-1 cells were exposed to 1,2-DCP at 0, 0.1, 0.2, 0.4, and 0.8 mM for 24 h, and cell viability was evaluated by MTS assay. Exposure of monocultured MMNK-1 cells to 1,2-DCP was associated with an increase in cell viability compared with the control group (Figure 1). On the other hand, MMNK-1 cells co-cultured with differentiated THP-1 cells showed no change in cell viability following exposure to 1,2-DCP, compared with the control (Figure 1). To examine whether the increase in cell viability in the monocultured MMNK-1 cells was related to increased cell proliferation, we performed cell count and BrdU assay. Cell count, but not BrdU assay, showed a significant increase in cell proliferation in the 1,2-DCP-exposed group compared with the control (Figure 2A–C).

### 3.2. 1,2-DCP Is Cytotoxic to Co-Cultured Cells but Not Monocultured Cells

**Figure 3:**
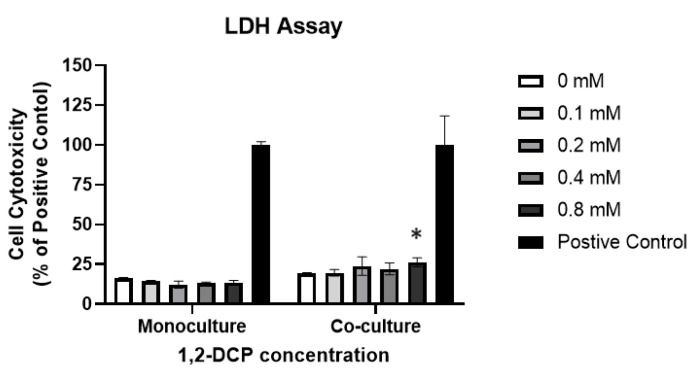
Cytotoxic effects of 1,2-DCP on monocultured MMNK-1 cholangiocytes and MMNK-1 cholangiocytes co-cultured with THP-1 macrophages, as assessed by the LDH Assay. MMNK-1 cholangiocytes or co-cultures of MMNK-1 cholangiocytes/THP-1 macrophages were exposed to 1,2-DCP at 0, 0.2, 0.4, and 0.8 mM for 24 h. Percentages are in relation to positive control. Data are mean ± SD, *n* = 3. * *p* < 0.05, compared with the control group (0 mM), by two-way ANOVA followed by Dunnett’s multiple comparison test. Two-way ANOVA showed a significant interaction between “1,2-DCP” and “macrophages”.

To determine the cytotoxic effect of 1,2-DCP, both types of cell cultures were exposed to 1,2-DCP at 0, 0.1, 0.2, 0.4, and 0.8 mM for 24 h. Lactate dehydrogenase (LDH), a cytoplasmic enzyme released into the extracellular environment upon cell membrane disruption during late-stage apoptosis or necrosis [43], was measured using the LDH cytotoxicity assay. Monocultured MMNK-1 cells exposed to 1,2-DCP showed no change in LDH release compared with the control group, while MMNK-1 cells co-cultured with differentiated THP-1 cells showed increased LDH cytotoxicity in the exposure group compared with the control group (Figure 3).

### 3.3. 1,2-DCP Causes DNA Damage in Co-Cultured Cells but Not Monocultured Cells

**Figure 4:**
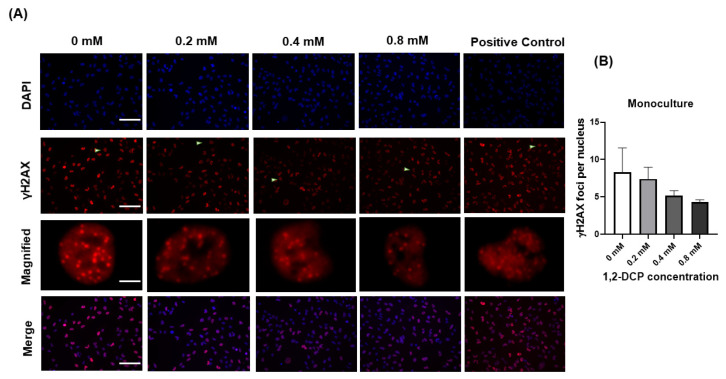
Effects of 1,2-DCP on DNA in monocultured MMNK-1 cholangiocytes, as assessed by γH2AX immunocytochemistry. Monocultured MMNK-1 cholangiocytes were exposed to 1,2-DCP at 0, 0.2, 0.4, and 0.8 mM for 24 h. For positive control, MMNK-1 cholangiocytes were exposed to 0.2 mM of H_2_O_2_ for 2 h. Cells were fixed with 4% PFA, permeabilized with 0.2% Triton-X, blocked with 1% BSA in PBST, and incubated with primary anti-γ-H2AX antibody overnight, then with secondary antibody. Nuclei were stained with DAPI and observed under a fluorescence microscope. Scale bar: 100 µm for all images except the magnified images (20 µm). Arrowheads: magnified images. (**A**) Photomicrographs of γH2AX immunostaining. (**B**) γH2AX foci count per nucleus. Data are mean ± SD, *n* = 3. compared with the control group (0 mM), by one-way ANOVA followed by Dunnett’s multiple comparison test.

**Figure 5:**
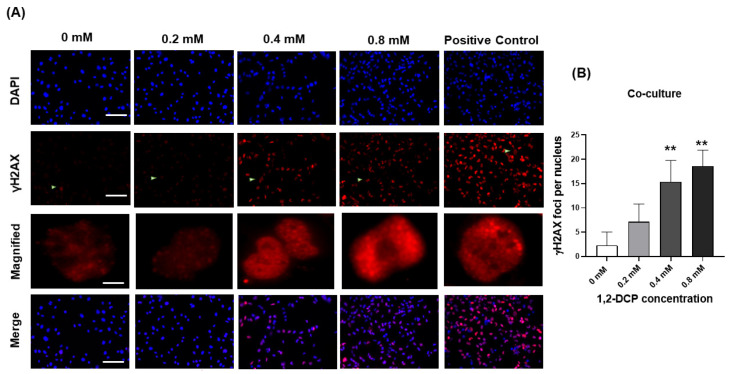
Effects of 1,2-DCP on DNA in MMNK-1 cholangiocytes co-cultured with THP-1 macrophages, as assessed by γH2AX immunocytochemistry. Co-cultures of MMNK-1 cholangiocytes and THP-1 macrophages were exposed to 1,2-DCP at 0, 0.2, 0.4, and 0.8 mM for 24 h. Positive control MMNK-1 cholangiocytes were exposed to 0.2 mM of H_2_O_2_ for 2 h. MMNK-1 cholangiocytes were fixed with 4% PFA, permeabilized with 0.2% Triton-X, blocked with 1% BSA in PBST, and incubated with primary anti-γ-H2AX antibody overnight, then with secondary antibody. Nuclei were stained with DAPI and examined by fluorescence microscopy. Scale bar: 100 µm for all images except the magnified images (20 µm). Arrowheads: magnified images. (**A**) Photomicrographs of γH2AX immunostaining. (**B**) γH2AX foci count per nucleus. Data are mean ± SD, *n* = 3., ** *p* < 0.01, compared with the control group (0 mM), by one-way ANOVA followed by Dunnett’s multiple comparison test.

In these experiments, we used two markers of DNA damage: γH2AX foci expression and Comet assay. Exposure of monocultured MMNK-1 cells to 1,2-DCP did not induce an increase in γH2AX foci expression, compared to the control (Figure 4A,B). In contrast, exposure of MMNK-1-THP-1 cells co-cultures induced an increase in γH2AX foci expression (Figure 5A,B). The foci found in the nuclei of monocultured MMNK-1 cells were typically discrete foci (Figure 4A), whereas those in co-cultured MMNK-1 cells were typically fine and homogeneously distributed within the nuclei (Figure 5A).

**Figure 6:**
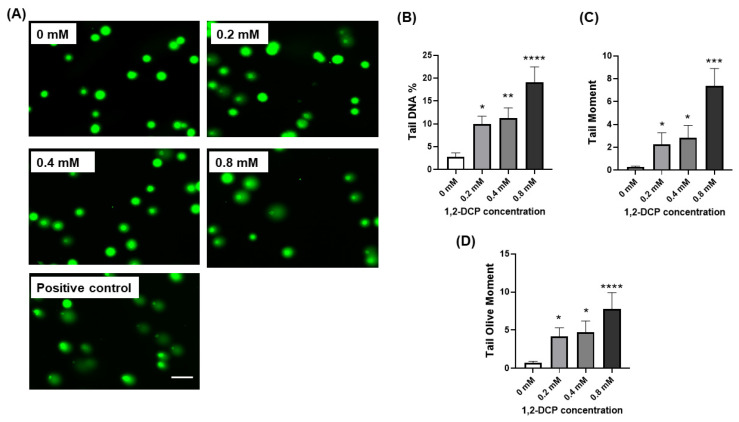
Effects of 1,2-DCP on DNA in MMNK-1 cholangiocytes/THP-1 macrophages, as assessed by Comet assay. Co-cultures of MMNK-1 cholangiocytes and THP-1 macrophages were exposed to 1,2-DCP at 0, 0.2, 0.4, and 0.8 mM for 24 h. For positive control, MMNK-1 cholangiocytes were exposed to 0.2 mM of H_2_O_2_ for 2 h. Alkaline Comet assay was performed. Scale bar: 100 µm. (**A**) Photomicrographs of Comet assay staining. (**B**) Tail DNA%. (**C**) Tail moment. (**D**) Tail olive moment. Data are mean ± SD, *n* = 3. * *p* < 0.05, ** *p* < 0.01, *** *p* < 0.001, **** *p* < 0.0001, compared with the control group (0 mM), by one-way ANOVA followed by Dunnett’s multiple comparison test.

Furthermore, quantitative assessment of DNA damage by the Comet assay using tail DNA%, tail moment, and tail olive moment showed an increase in DNA damage in the exposure group compared with the control (Figure 6B–D).

### 3.4. 1,2-DCP Enhances ROS Production in Co-Cultured Cells but Not Monocultured Cells

Previous studies found that exposure to 1,2-DCP depletes glutathione in rats [49], rationalizing the hypothesis that exposure to 1,2-DCP increases ROS level through depletion of glutathione. DCFDA assay is commonly used to detect ROS in cells when they are oxidized to a fluorescence dye (DCF), which is detected at excitation wavelength 485 nm and emission wavelength 535 nm using a fluorescent plate reader [50]

**Figure 7:**
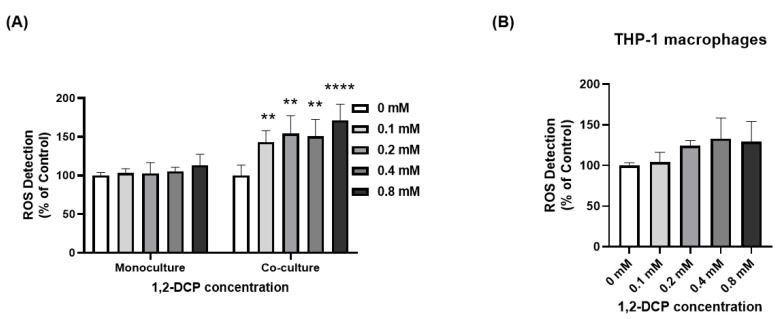
Effects of 1,2-DCP on ROS production, as assessed by DCFDA assay. (**A**) monocultures of MMNK-1 cells and MMNK-1 cells co-cultured with THP-1 macrophages. (**B**) THP-1 macrophages exposed to 1,2-DCP at 0, 0.1, 0.2, 0.4, and 0.8 mM for 24 h. Percentages are in relation to 0 mM 1,2-DCP group. Data are mean ± SD, *n* = 3. ** *p* < 0.01, **** *p* < 0.0001 compared with the control group (0 mM), by two-way ANOVA (**A**) and one-way ANOVA (**B**) followed by Dunnett’s multiple comparison test. Two-way ANOVA showed a significant interaction between “1,2-DCP” and “macrophages”.

Exposure of monocultured MMNK-1 cells to 1,2-DCP was not associated with significant change in ROS production compared to the control. However, exposure of MMNK-1-THP-1 co-cultured cells was associated with increased ROS production compared with the control group (Figure 7A). Interestingly, 1,2-DCP did not increase ROS production in differentiated THP-1 cells (Figure 7B). The multiple regression model showed significant interaction between macrophage factor and 1,2-DCP exposure level (Table 2), suggesting different dose-response curves in the presence of macrophages.

### 3.5. 1,2-DCP Induces TNF-α and IL-1β in THP-1 Macrophages

**Figure 8:**
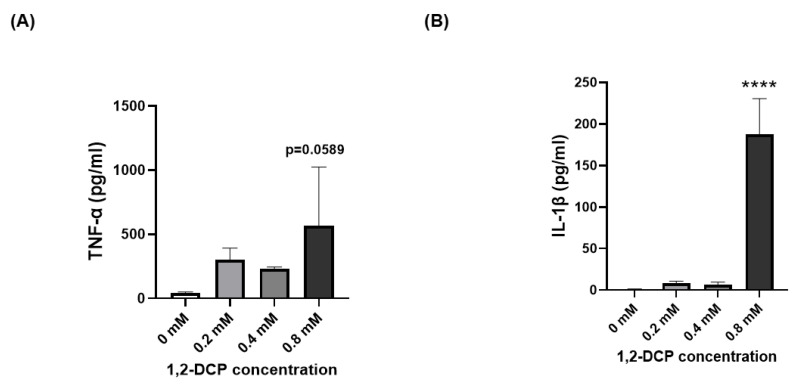
Effects of 1,2-DCP on TNF-α and IL-1β protein expression in THP-1 macrophages. THP-1 macrophages were exposed to 1,2-DCP at 0, 0.2, 0.4, and 0.8 mM for 24 h, and cell culture supernatants were collected. Protein expression was measured by ELISA. (**A**) TNF-α, (**B**) IL-1β. Data are mean ± SD, *n* = 3. **** *p* < 0.0001 compared with the control group (0 mM), by one-way ANOVA followed by Dunnett’s multiple comparison test.

To examine the involvement of pro-inflammatory mediators in ROS over-production in the cell co-cultures, we assessed TNF-α and IL-1β protein expression by ELISA. Exposure to 1,2-DCP for 24 h increased TNF-α and IL-1β protein expression in differentiated THP-1 cells compared to the control group, and the increase in IL-1β protein expression was significant (Figure 8A,B).

## 4. Discussion

The present study investigated the role of macrophages in 1,2-DCP-induced cell proliferation/cytotoxicity, DNA damage, and ROS production. The results showed that the deleterious effects of 1,2-DCP on cytotoxicity, DNA damage, and ROS production of cholangiocytes were enhanced in the presence of THP-1 macrophages compared to monocultures of cholangiocytes.

In this study, monocultures of cholangiocytes or co-cultures of cholangiocytes with macrophages were exposed to 1,2-DCP. Macrophages were used as inflammatory cells in the co-culture model based on the reported importance of hepatic macrophages, including residential Kupffer cells and infiltrating monocyte-derived macrophages, in inflammation and fibrosis of the liver [51].

The results of the MTS cell viability assay (Figure 1) and cell count (Figure 2C) showed that 1,2-DCP induced proliferation of monocultured MMNK-1 cells but not MMNK-1 cells co-cultured with THP-1 macrophages. This finding is in agreement with our previous study that showed exposure of monocultured MMNK-1 cells to 1,2-DCP increased MTS cell viability [27]. However, in this study, cell proliferation assessed by BrdU assay did not show a significant increase in cell proliferation (Figure 2B). This could be due to the fact that BrdU assay measures cells undergoing DNA replication within the period of time of the application of the reagents, whilst cell count detects the overall number of cells [52]. Furthermore, the increased viability as measured by the MTS (5-(3-carboxymethoxyphenyl)-2-(4,5-dimethyl-thiazoly)-3-(4-sulfophenyl) tetrazolium) assay indicates increased metabolism, which is able to convert MTS into colored formazan, which is measured at absorbance 490 nm [53]. Taken together with the data of the cell count in mono-cultured MMNK-1 cells, the results of the present study on MTS cell viability in monocultured MMNK-1 cells exposed to 1,2-DCP suggest increased proliferation.

It is known that proliferating cells have less time to repair damaged DNA [28,54]. Therefore, if cellular division occurs before DNA repair systems can act, then the injury becomes permanent and irreversible [34,55]. Increased cell proliferation, therefore, increases the chances of spontaneous mutations. DNA damage and/or mutation affecting genes responsible for neoplastic development can increase the probability of carcinogenesis [28,35,56]. The increased cell proliferation of cholangiocytes observed in our study may explain the biliary hyperplasia observed previously in non-cancerous hepatic tissues from cholangiocarcinoma in the 1,2-DCP-exposed workers [4], as well as increased cholangiocyte proliferation in mice exposed for 4 weeks to 1,2-DCP by inhalation [57].

Exposure of MMNK-1 cells co-cultured with THP-1 macrophages to 1,2-DCP for 24 h was associated with increased DNA damage in both tests employed in this study for detection of DNA damage. The results of Comet assay showed that 1,2-DCP increased dose-dependently tail DNA%, tail moment, and olive tail moment (Figure 6B–D), as well as γH2AX foci expression in cholangiocytes co-cultured with macrophages (Figure 5B), but not in MMNK-1 cells cultured alone (Figure 4B), suggesting that the inflammatory environment enhances the DNA damage in cholangiocytes exposed to 1,2-DCP. This is in agreement with our previous studies, which showed increased tail DNA% and tail moment, and over-expression of γH2AX foci in MMNK-1/macrophage co-cultures exposed to 1,2-DCP [27,58]. Comet assay measures both single- and double-strand breaks (SSB and DSB) of DNA damages, whilst γH2AX expression measures DSBs. γH2AX foci are formed following phosphorylation of the Ser-139 residue of the histone variant H2AX, representing an early cellular response to the induction of DNA double-strand breaks [59]. DNA damage has been established as the main cause for chemical carcinogenesis [28]. DSBs are the most dangerous form of DNA lesions, which, if left unrepaired, may lead to chromosome aberrations, genomic instability, or cell death [60]. Furthermore, accumulation of chromosome aberrations or mutation over time could lead to cancer [61]. Our finding of DNA damage is in agreement with increased γH2AX expression in invasive carcinoma, BilIN, and IPNB and at sites of non-neoplastic biliary epithelium of the large bile duct in cholangiocarcinoma specimens obtained from workers exposed to 1,2-DCP [9]. Thus, macrophages appear to enhance the DNA-damaging effect of 1,2-DCP in cholangiocytes.

Our results differ from those of a recent study, which showed 1,2-DCP-induced over-expression of γH2AX in monocultured MMNK-1 cells [62]. This could be due to the relatively high concentrations of 1,2-DCP used in the above study. Our study used a more realistic exposure concentration range of 1,2-DCP relative to the blood concentrations found in the color proof-printing Japanese workers [3].

Interestingly, exposure to 1,2-DCP increased ROS levels in MMNK-1 cells co-cultured with macrophages, but not in monocultured MMNK-1 cells or THP-1 macrophages (Figure 7A,B). ROS are known as inducers of cell death [63], therefore this could explain the increased cytotoxicity of the co-cultured cells compared to the monocultures (Figure 3, Table 2). Furthermore, the results also suggest that ROS are generated within cholangiocytes through intrinsic mechanisms set by the macrophages, given the minimal increase in ROS production in macrophages alone. Although activated inflammatory cells are known to produce large amounts of ROS at the site of inflammation [64], our results showed a lesser level of increase in ROS in macrophages than in cholangiocytes co-cultured with macrophages following exposure to 1,2-DCP (Figure 7A,B), suggesting the increase in ROS in cholangiocytes is not derived mainly from ROS in macrophages. Previous studies showed enhanced toxicity of certain chemicals in the presence of inflammatory cells [23], which was mediated through ROS production [30,31,32].

Furthermore, 24-h exposure to 1,2-DCP increased the expression levels of TNF-α marginally and IL-1β significantly in THP-1 macrophages (Figure 8A,B). It is well established that TNF-α and IL-1β are key cytokines secreted by macrophages during inflammation [65], indicating that exposure of macrophages to 1,2-DCP induces an inflammatory response. This is consistent with our previous study, which reported increased TNF-α protein expression in the culture medium of THP-1 macrophages exposed to 1,2-DCP [58]. Furthermore, inflammation is known to enhance the production of ROS [66], and more specifically, both TNF-α and IL-1β are known to stimulate the production of ROS in cells [67,68,69,70].

However, 1,2-DCP is known to be metabolized by CYP2E1, and one product of such metabolism is ROS [71,72,73]. 1,2-DCP has also been shown to induce mitochondrial dysfunction in the liver of mice, which could enhance ROS generation and the development of oxidative stress [74]. Previous studies indicated that exposure of rats to 1,2-DCP resulted in glutathione depletion, while an in vitro study found spontaneous conjugation of 1,2-DCP with glutathione [49,75]. Glutathione depletion could increase ROS levels in the cells. It is therefore likely that additional sources contribute to the high ROS levels generated in co-cultures of cholangiocytes compared to monocultures (Figure 7A). Further research is needed to investigate the role of mitochondrial dysfunction and CYP2E1 in ROS generation in a co-culture model.

The high ROS levels could account for the increased DNA damage observed in 1,2-DCP-exposed co-cultured MMNK-1 cells (Figure 5 and Figure 6). Under normal physiological conditions, small increases in ROS levels activate various signaling pathways to initiate biological processes. However, high increases of ROS levels lead to oxidative stress that results in damage to DNA, proteins and lipids [23,29,31]. Over time, this could result in genomic instability, diseases, and cancer [76].

To the best of our knowledge, this study is the first to demonstrate the increase production of ROS in cholangiocytes co-cultured with THP-1 macrophages when exposed to 1,2-DCP. Moreover, this study demonstrates the cytotoxic effects of 1,2-DCP on cholangiocytes in the presence of macrophages using a 1,2-DCP concentration range that is commensurate with the exposure levels of 1,2-DCP among the workers who developed cholangiocarcinoma.

Nonetheless, some limitations are to be noted. First, the present study investigated mechanisms of development of 1,2-DCP-induced cholangiocarcinoma using immortalized cholangiocyte cell line-MMNK-1 in the presence or absence of macrophages. Further studies using other cholangiocyte cell lines such as NHC cells or H69 cells to replicate the primary findings of the present study is warranted. Secondly, the detection of cytotoxicity using LDH assay includes cell death resulting from both apoptosis and necrosis. It is difficult to attribute the result of LDH cytotoxicity to only apoptosis or necrosis. Future research is therefore needed to explore the occurrence of apoptosis using a caspase 3/7 assay. As γH2AX foci formation has also been associated with telomere shortening in senescent cells [77], further studies are warranted to determine if cholangiocytes or macrophages exposed to 1,2-DCP show increased cellular senescence.

## 5. Conclusions

We have demonstrated in the present study that the presence of macrophages aggravated the effects of 1,2-DCP on cytotoxicity, DNA damage, and ROS production in cholangiocytes. Exposure to 1,2-DCP increased cholangiocyte cell proliferation and proinflammatory cytokines in macrophages. The results suggest that the presence of inflammatory cells play a role in the cytotoxic and DNA-damaging effects of 1,2-DCP. This may be the underlying mechanism of 1,2-DCP-induced carcinogenesis in cholangiocytes, through ROS production and inflammatory response.

## Figures and Tables

**Table 1:** Seeding densities for various research methods used in the study.

Research Methods	Cells Seeded (Cells/Well)			
	Monoculture	Co-Culture		Monoculture
	MMNK-1 Cells	MMNK-1 Cells	THP-1 Cells	THP-1 Cells
MTS Assay	1 × 10^4^ (96-well plate)	3 × 10^4^ (24-well plate)	6 × 10^4^ (24-well inserts)	-
BrdU	6 × 10^4^ (12-well plate)	-	-	-
Cell count	3 × 10^4^ (24-well plate)	-	-	-
LDH Assay	5 × 10^3^ (96-well plate)	3 × 10^4^ (24-well plate)	6 × 10^4^ (24-well inserts)	-
ROS detection	1.3 × 10^4^ (96-well plate)	1 × 10^5^ (24-well plate)	2 × 10^5^ (24-well inserts)	3 × 10^4^ (96-well plate)
γH2AX	2.5 × 10^4^ (24-well plate)	2.5 × 10^4^ (24-well plate)	5 × 10^4^ (24-well inserts)	-
Comet Assay	-	1.5 × 10^5^ (6-well plate)	3 × 10^5^ (6-well inserts)	-
ELISA	-	-	-	2 × 10^5^ (96-well plate)

**Table 2:** Effects of 1,2-DCP on cytotoxicity and oxidative stress.

	Model	1,2-DCP Concentration (mM)					Simple Regression	Multiple Regression (*p* Value)		
		0	0.1	0.2	0.4	0.8	Effect of 1,2-DCP (*p* Value)	1,2-DCP and Macrophage Interaction	Effect of 1,2-DCP	Macrophage Factor
ROS production (%)	*Monocultures*	100 ± 4	103 ± 6	103 ± 14	106 ± 5	113 ± 14	16 (0.065)	51 (0.03)	-	-
	*Co-cultures*	100 ± 9	143 ± 4 *	154 ± 3 *	151 ± 1 *	171 ± 2 *	67 (0.007)			
MTS Assay (%)	*Monocultures*	100 ± 21	108 ± 45	165 ± 14 *	177 ± 8 *	163 ± 10 *	78 (0.03)	−56 (0.18)	78 (0.012)	−14 (0.24)
	*Co-cultures*	100 ± 6	137 ± 4	136 ± 17	141 ± 22	131 ± 8	22 (0.42)			
LDH Assay (%)	*Monocultures*	16 ± 3	14 ± 3	12 ± 14	13 ± 3	14 ± 7	−2 (0.20)	10 (0.006)	-	-
	*Co-cultures*	19 ± 1	20 ± 2	24 ± 6	22 ± 4	26 ± 3 *	8 (0.018)			

Data are mean ± SD. * *p* < 0.05, compared with the corresponding control by two-way ANOVA followed by Dunnett’s multiple comparison test for ROS production, MTS Assay, and LDH Assay (*n* = 3). Simple regression analysis and test for interaction in multiple regression model were conducted for ROS production, MTS Assay, and LDH Assay. Values were expressed as percentages in relation to 0 mM 1,2-DCP group for ROS production and MTS assay, while LDH assay values were expressed as percentages in relation to the positive control treated with lysis buffer. Since a significant interaction was found for ROS production and LDH Assay, the effect of 1,2-DCP in the multiple regression model could not be tested.

## Supplementary Materials

The following are available online at https://www.mdpi.com/article/10.3390/toxics9060128/s1. Figure S1: Illustration of co-culture model. Table S1: Protein concentration values for proinflammatory cytokines expression analysis by ELISA.
