# Supplementary material for: Role of Macrophages in Cytotoxicity, Reactive Oxygen Species Production and DNA Damage in 1,2-Dichloropropane-Exposed Human Cholangiocytes In Vitro
Source: Toxics. 2021 Jun 1;9(6):128. doi: 10.3390/toxics9060128 (PMC8228395; doi:10.3390/toxics9060128)
Supplement: Supplementary file 1 [file toxics-09-00128-s001.zip › toxics-1211472-supplementary.pdf]

Supplementary Materials: Role of Macrophages in Cytotoxicity, Reactive Oxygen Species Production and DNA Damage in 1,2-Dichloropropane-Exposed Human Cholangiocytes In Vitro

Abigail Ekuban, Cai Zong, Frederick Adams Ekuban, Yusuke Kimura, Ryoya Takizawa, Kota Morikawa, Kazuo Kinoshita, Sahoko Ichihara, Seiichiroh Ohsako and Gaku Ichihara

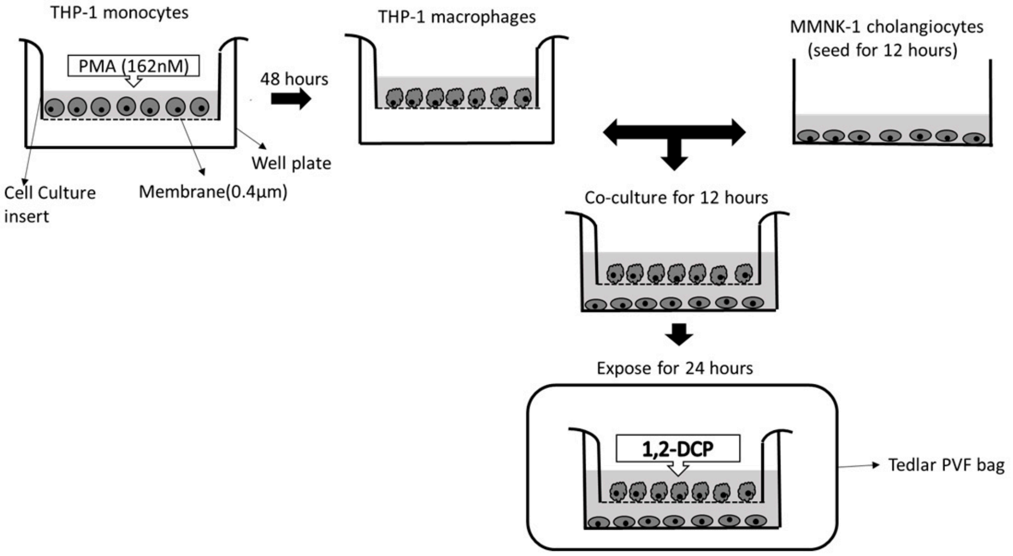

Figure S1. Illustration of the co-culture model.THP-1 monocytes were differentiated into macrophages by PMA at 162nM concentration by seeding into cell culture inserts (pore size membrane: 0.4 μm) for 48 hours. MMNK-1 cholangiocytes were seeded in well plate for 12 hours and then combined with THP-1 macrophages. MMNK-1 cholangiocytes and THP-1 macrophages were co-cultured for 12 hours and then exposed to different concentrations of 1,2-DCP for 24 hours. The MMNK-1 cholangiocytes and THP-1 macrophages were placed into Tedlar polyvinyl fluoride (PVF) gas sampling bags to prevent evaporation of 1,2-DCP and incubated at 37°C.

Table S1. Protein concentration values for proinflammatory cytokines expression analysis by ELISA.

1,2-DCP Concentration(mM)	Protein Concentration (pg/ml)	
	**TNF-α**	**IL-1β**
0	41±10	0.9±0.7
0.2	301±91	8±2
0.4	232±14	6±3
0.8	568±457	187±43*

Protein concentrations are expressed as mean ±SD, *$p<0.05$, compared with corresponding control by one-way ANOVA followed by Dunnett's test multiple comparison test.
